# The Impact of Mini‐Screws and Micro‐Implants on Orthodontic Clinical Outcomes: An Umbrella Meta‐Analysis

**DOI:** 10.1002/cre2.70220

**Published:** 2025-09-08

**Authors:** Abdolreza Jamilian, Helen Jamloo, Kurosh Majidi, Meysam Zarezadeh

**Affiliations:** ^1^ The City of London Dental School University of Greater Manchester Bolton UK; ^2^ Orthodontic Department, Dental School, Tehran Medical Sciences Islamic Azad University Tehran Iran; ^3^ School of Dentistry Tabriz University of Medical Sciences Tabriz Iran; ^4^ Department of Orthodontics Smile Tailor Private Practice London UK; ^5^ Drug Applied Research Center Tabriz University of Medical Sciences Tabriz Iran; ^6^ Women's Reproductive Health Research Center Tabriz University of Medical Sciences Tabriz Iran

**Keywords:** micro‐implants, mini‐screws, orthodontics, umbrella meta‐analysis

## Abstract

**Objectives:**

This umbrella meta‐analysis aimed to answer the clinical question: Do mini‐screws and micro‐implants improve specific orthodontic outcomes such as intermolar width, interpremolar width, suture expansion, molar movement, and skeletal width compared to conventional anchorage methods?

**Materials and Methods:**

A systematic search was performed in PubMed, Scopus, ISI Web of Science, and Google Scholar up to October 2024. Systematic reviews and meta‐analyses on mini‐screws and micro‐implants in orthodontic treatment were included. Methodological quality was assessed using AMSTAR 2, and a random‐effects model was used to calculate effect sizes (ESs) and 95% confidence intervals (CIs). Heterogeneity was evaluated using the *I*² statistic and Cochrane's Q‐test, with subgroup and sensitivity analyses conducted to identify sources of heterogeneity.

**Results:**

Eleven meta‐analyses comprising 50 data sets were included. The results indicated that mini‐screws significantly increased intermolar width (ES: 2.61 mm, 95% CI: 0.29–4.92, *p* = 0.02) and skeletal width (ES: 3.33 mm, 95% CI: 1.37–5.29, *p* = 0.001). However, no significant impact was found on interpremolar width or alveolar width before and after MARPE. Micro‐implants significantly reduced molar movement (ES: −1.13 mm, 95% CI: −1.99 to −0.26, *p* = 0.01). Substantial heterogeneity was noted across several outcomes (*I*² > 50%), which persisted despite subgroup analyses.

**Conclusion:**

Mini‐screws and micro‐implants enhance skeletal and intermolar width and control molar movement in orthodontic treatment. However, their effects on other outcomes are inconsistent, warranting further high‐quality studies to strengthen clinical recommendations.

**Trial Registration:** PROSPERRO (CRD42023447137).

## 1 Introduction

Orthodontics is a dental specialty dedicated to diagnosing, preventing, and treating dental and facial irregularities. It involves various techniques and tools to address issues such as crooked or crowded teeth, overbites, underbites, and other types of malocclusions (Ghodasra and Brizuela 2023). Overall, orthodontic treatment can help to improve the appearance and function of the teeth and jaw, leading to better overall oral health and a more confident smile.

Mini‐screws and micro‐implants are orthodontic anchorage devices designed to provide support and enhance stability during treatment. These small, screw‐like titanium alloy structures are surgically inserted into the bone to serve as secure anchor points for tooth repositioning (Singh et al. 2010). The use of mini‐screws and micro‐implants depends on the specifics of each case and the orthodontist's treatment goals. Overall, these devices provide essential anchorage support, enhancing tooth movement precision and improving treatment outcomes (Justens and De Bruyn 2008). Mini‐screws are larger than micro‐implants and are typically used in cases requiring stronger anchorage, such as severe crowding or closing interdental spaces. In contrast, micro‐implants, being smaller, are suited for situations requiring minimal anchorage, such as mild to moderate crowding or the movement of a single tooth (Sharma and Sangwan 2014). Compared to other orthodontic treatment methods, mini‐screws and micro‐implants offer several advantages. They provide greater control and precision in tooth movement, potentially shortening treatment time and improving outcomes. Additionally, they reduce the need for external devices, such as headgear, which can be uncomfortable and inconvenient for patients (Keser and Naini 2022). Traditional orthodontic methods, such as braces and clear aligners, also use anchorage to facilitate tooth movement. However, they rely on surrounding teeth as anchor points, which can limit the extent of movement. In contrast, mini‐screws and micro‐implants offer a more stable and direct anchorage, enabling more precise and efficient tooth repositioning (Keser and Naini 2022). In summary, mini‐screws and micro‐implants are important tools in the orthodontic toolkit, providing anchorage support and allowing for greater control and precision in tooth movement during orthodontic treatment (Wiechmann et al. 2007).

Numerous meta‐analyses have investigated the effects of mini‐screws and micro‐implants on various orthodontic outcomes, including premolar and intermolar width, skeletal width expansion, alveolar width changes during MARPE, tooth movement, posterior nasal spine position, and treatment duration. However, the results remain inconsistent. While some studies highlight beneficial effects, others report no statistically significant impact. Additionally, substantial heterogeneity across findings has contributed to uncertainty regarding the overall effectiveness of these interventions in orthodontic procedures. Therefore, this umbrella meta‐analysis focuses on the mechanical effects of mini‐screws and micro‐implants on specific orthodontic measures such as intermolar and interpremolar expansion and molar anchorage stabilization, aiming to synthesize results for these well‐defined outcomes.

## 2 Methods

This umbrella review was conducted in accordance with the Preferred Reporting Items for Systematic Reviews and Meta‐Analyses (PRISMA) 2020 guidelines (Page et al. 2021). A completed PRISMA checklist is provided in the Supporting Material.

### 2.1 Literature Search and Selection Criteria

A comprehensive examination of systematic reviews and meta‐analyses was undertaken in accordance with standard procedures of preferred reporting items for systematic reviews and meta‐analyses (Aromataris et al. 2015; Ioannidis 2009). A comprehensive search was conducted in PubMed, Scopus, Web of Science, and Google Scholar to identify relevant studies published up to October 2024. The search pattern used to search the databases is provided in the Supporting File S2. Additionally, the reference lists of all included articles were manually screened to identify any additional eligible studies. The search was limited to studies only in the English language.

### 2.2 Inclusion and Exclusion Criteria

Two independent reviewers (A.J., K.M.) screened the titles and abstracts based on the PICO framework. The population included patients undergoing fixed orthodontic treatments, such as those involving braces or rapid maxillary expansion, where skeletal anchorage was indicated. The intervention of interest was the use of mini‐screws or micro‐implants as temporary anchorage devices to support or enhance tooth movement. Comparator groups consisted of patients treated with conventional anchorage methods. The primary outcomes assessed were changes in interpremolar and intermolar width, skeletal and alveolar width before and after mini‐screw‐assisted rapid palatal expansion, molar movement, posterior nasal spine changes, and overall treatment duration. These outcomes were evaluated using methods such as digital dental models, cone beam computed tomography, lateral cephalometric radiographs, and clinical records, as reported in the original meta‐analyses.

This umbrella meta‐analysis included only meta‐analyses that synthesized data from randomized controlled trials and/or controlled clinical trials assessing the efficacy of mini‐screws and micro‐implants on orthodontic treatment outcomes and provided pooled effect sizes with corresponding confidence intervals. Studies were excluded if they were in vitro, in vivo, or ex vivo in design, if they were observational in nature, or if they lacked sufficient information—such as missing pooled effect sizes or confidence intervals—required for synthesis in an umbrella meta‐analysis. Only articles published in the English language were included, which may introduce potential language‐based publication bias. Future reviews may benefit from the inclusion of studies published in other languages to provide a more comprehensive synthesis of the available evidence.

### 2.3 Data Extraction

Two reviewers extracted the following data independently: Publication year, sample size, study location, effect sizes, and their respective CIs for width at the first premolars, dental inter‐molar width increase, skeletal width increase, alveolar width before and after MARPE, tooth movement, PNS, and treatment duration were extracted from the selected meta‐analyses. Any disagreements were resolved by discussion with the third reviewer (M.Z.).

### 2.4 Risk of Bias Assessment

Two reviewers (A.J., K.M.) independently evaluated the eligible papers' risk of bias using the AMSTAR 2 questionnaire (Shea et al. 2007). There are 16 questions in the AMSTAR questionnaire about the systematic reviews' methodological quality, and respondents can select “yes,” “no,” “cannot answer,” or “not applicable” as their response option.

### 2.5 Data Synthesis and Statistical Analysis

To determine the overall effect sizes for each meta‐analysis, random‐effects model with restricted maximum likelihood method (REML) was employed. The *I*² statistic and Q‐test *p*‐value were interpreted as indicators of heterogeneity rather than strict thresholds. Contextual factors, such as differences in populations and interventions, were considered. *I* ^2^ value > 50% or *p* < 0.1 for the Q‐test was considered as a statistically significant between‐study heterogeneity (Higgins and Thompson 2002). When data were available, subgroup analyses were carried out based on the number of included studies and intervention type to identify likely causes of heterogeneity. The dependence of the total effect size on a specific meta‐analysis was discovered using a one‐study removal sensitivity analysis. Stata version 16 (Stata Corporation, College Station, TX, US) was used for the meta‐analysis. A two‐sided *p*‐value of 0.05 was regarded as statistically significant unless otherwise stated. If the number of observations for each meta‐analysis was less than ten, small study effect analyses and visual inspection of funnel plot was not performed (Higgins et al. 2022).

## 3 Result

### 3.1 Study Selection

**Figure 1:**
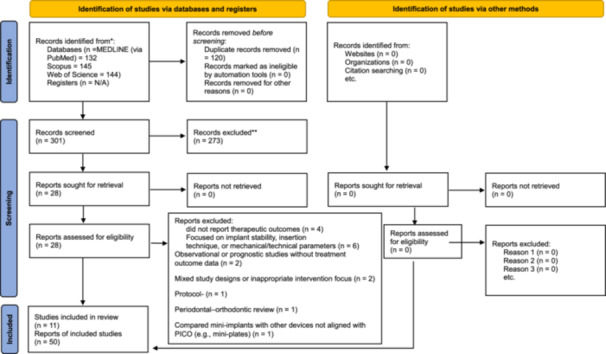
PRISMA 2020 flow diagram summarizing study selection for this umbrella review. After deduplication, 301 records were screened; 28 full texts were assessed; 11 studies (11 reports) were included. Note: Across these 11 meta‐analyses, we extracted 50 independent outcome data sets, which are described in the Results and the Supporting Materials, not counted as reports in PRISMA.

The present study incorporated eleven meta‐analyses of controlled trials encompassing a total of fifty data sets published between 2017 and 2023. Following the revision and expansion of our search strategy, a total of 421 records were identified across PubMed, Scopus, and Web of Science. After removing 120 duplicate records, 301 unique articles remained for screening. Upon reviewing titles and abstracts, 273 articles were excluded due to irrelevance. The full texts of 28 articles were assessed for eligibility, of which 17 were excluded for reasons including lack of relevant data (*n* = 9), observational design/no pooled outcomes (*n* = 3), biomechanical focus (e.g., cortical thickness, insertion method, thread design) (*n* = 3) and protocol design (*n* = 1). Ultimately, 11 meta‐analyses, comprising 50 independent data sets, met the inclusion criteria and were included in this umbrella review. In addition to database searches, the PROSPERO register was screened to identify any ongoing or recently completed systematic reviews or meta‐analyses related to the use of mini‐screws and micro‐implants in orthodontics. Although no eligible studies were identified through this source, its inclusion aimed to enhance the comprehensiveness of the search strategy and reduce the risk of publication bias. Figure 1 displays the updated PRISMA flow diagram outlining the study selection process.

### 3.2 Study Characteristics

Table 1 provides a summary of the key characteristics of the studies that were included in the analysis. For meta‐analyses that reported multiple independent effect sizes for different outcomes, each data set was treated as a separate entry in the analysis and presented individually in Table 1, in accordance with umbrella review methodology. four data sets pertaining to the impact of mini‐screws on inter‐premolar width (Siddhisaributr et al. 2022; Bi and Li 2022), two data sets examining treatment duration (Alharbi et al. 2019; Antoszewska‐Smith et al. 2017), eight data sets investigating inter‐molar width (Siddhisaributr et al. 2022; Bi and Li 2022; Huang et al. 2022; Kapetanović et al. 2021), two data sets exploring skeletal width increase (Siddhisaributr et al. 2022; Kapetanović et al. 2021), two data sets analyzing alveolar width before and after MARPE (Huang et al. 2022), three data sets assessing tooth movement (Antoszewska‐Smith et al. 2017; Huang et al. 2022; Dos Santos et al. 2020), and two data sets examining mid‐palatal suture expansion (Siddhisaributr et al. 2022; Bi and Li 2022). Furthermore, within the realm of literature pertaining to micro‐implants, four distinct data sets pertain to the movement of molars (Peng et al. 2023; Liu et al. 2020; Xu and Xie 2017). Data sets that met the predefined inclusion criteria were incorporated into the quantitative meta‐analysis. Data sets that did not meet these criteria—due to high heterogeneity or incomplete reporting—were not included in the quantitative synthesis but were considered in a qualitative manner to ensure a comprehensive understanding of the evidence landscape. Regarding the quality of the meta‐analyses incorporated in our study, it was determined that three of them exhibited a high level of quality (Siddhisaributr et al. 2022; Kapetanović et al. 2021; Dos Santos et al. 2020), two were deemed to possess a moderate level of quality (Alharbi et al. 2019; Antoszewska‐Smith et al. 2017), three were found to have a low level of quality (Peng et al. 2023; Sosly et al. 2020; Li et al. 2011) and three studies have Critically Low quality (Bi and Li 2022; Huang et al. 2022; Xu and Xie 2017). The majority of the meta‐analyses included in this umbrella study failed to provide a comprehensive explanation of the specific characteristics of the controlled trials that met the inclusion criteria, as well as the process used to assess the quality of the studies included in their analysis. This lack of detail has the potential to impact the overall quality of the meta‐analyses, as indicated in Table 2.

**Table 1:** Study characteristics of included studies.

First author	Year	Included studies (*n*)	Participants (*n*)	Age	Gender	Key findings	Outcome	Quality assessment
Alharbi (a) (Alharbi et al. 2019)	2019	7	241	14.22 to −22.4	NR	Mini screws preserved ≈2 mm more anchorage than headgear/TPA during en‐masse retraction; limited data on time/adverse events	Anchorage loss measured as mesial displacement of molars (mm)	Yes (Cochrane)
Alharbi (b) (Alharbi et al. 2019)	2019	3	105	14.22 to −22.5	NR		Overall orthodontic treatment duration (months)	Yes (Cochrane)
Guang Bi (a) (Bi and Li 2022)	2022	2	110	8 to −13	NR	Greater palatal suture opening (ANS/PNS/1PM/1 M), ↑ palatal width at 1 M, and ↓ buccal inclination of premolars/molars with MARME.	Mid‐palatal suture expansion (mm) measured at the anterior nasal spine (Anterior nasal spine expansion (mm))	Yes (Cochrane)
Guang Bi (b) (Bi and Li 2022)	2022	2	110	8 to −13	NR		Mid‐palatal suture expansion (mm) at the level of the first premolars	Yes (Cochrane)
Guang Bi (c) (Bi and Li 2022)	2022	2	110	8 to −13	NR		Mid‐palatal suture expansion (mm) at the level of the first molars	Yes (Cochrane)
Guang Bi (d) (Bi and Li 2022)	2022	2	110	8 to −13	NR		Mid‐palatal suture expansion (mm) measured at the posterior nasal spine (Posterior nasal spine expansion (mm))	Yes (Cochrane)
Guang Bi (e) (Bi and Li 2022)	2022	2	83	≥ 11	NR		Palatal width at the first molars before and after expansion (mm)	Yes (Cochrane)
Guang Bi (f) (Bi and Li 2022)	2022	5	210	≥ 11	NR		Transverse width measured at the first premolars (mm)	Yes (Cochrane)
Guang Bi (g) (Bi and Li 2022)	2022	4	168	≥ 11	NR		Buccal inclination of first premolars (degrees)	Yes (Cochrane)
Guang Bi (h) (Bi and Li 2022)	2022	5	218	≥ 11	NR		Buccal inclination of first molars (degrees)	Yes (Cochrane)
Antoszewska‐Smith (a) (Antoszewska‐Smith et al. 2017)	2017	13	596	19.43	f/m	Mini screws/TISADs achieved ~1.86 mm greater anchorage preservation vs conventional methods; overall better anchorage control.	Mesial molar movement (mm) due to anchorage loss	Yes (Cochrane)
Antoszewska‐Smith (b) (Antoszewska‐Smith et al. 2017)	2017	5	220	17.5	f/m		Tipping angle of molars (degrees)	Yes (Cochrane)
Antoszewska‐Smith (c) (Antoszewska‐Smith et al. 2017)	2017	12	538	19.5	f/m		Amount of incisor retraction (mm)	Yes (Cochrane)
Antoszewska‐Smith (d) (Antoszewska‐Smith et al. 2017)	2017	11	447	19.5	f/m		Tipping angle of incisors (degrees)	Yes (Cochrane)
Antoszewska‐Smith (e) (Antoszewska‐Smith et al. 2017)	2017	11	191	22.5	f/m		Overall orthodontic treatment duration (months)	Yes (Cochrane)
Kapetanović (a) (Kapetanović et al. 2021)	2021	7	129	21.4	f/m	High success rate (~92.5%); ↑ skeletal width and ↑ dental intermolar width; noted dental tipping, ↓ buccal bone thickness/height; expansion time 20–126 days.	Skeletal transverse width increase (mm)	Yes (Cochrane)
Kapetanović (b) (Kapetanović et al. 2021)	2021	5	76	21.2	f/m		Change in dental intermolar width (mm)	Yes (Cochrane)
Oliveira dos Santos (Dos Santos et al. 2020)	2020	5	182	22.4	f/m	No significant acceleration of tooth movement; no effect on anchorage loss, root resorption, or periodontal health; mild pain/QoL impact; Propel ≈ other minis crews.	Overall linear tooth movement (mm)	Yes (Cochrane)
Sosly (a) (Sosly et al. 2020)	2020	5	130	NR	NR	Mini screws gave greater deep‐bite reduction (SMD − 0.48), less molar extrusion (SMD − 0.86), more incisor intrusion (SMD − 0.95); no significant difference in root resorption.	Vertical bite closure (mm) from deep‐bite correction	Yes (Cochrane)
Sosly (b) (Sosly et al. 2020).	2020	5	121	NR	NR		Incisor intrusion measured as vertical distance from incisal edge to palatal plane (Cr‐PP, mm)	Yes (Cochrane)
Sosly (c) (Sosly et al. 2020).	2020	6	162	NR	NR		Vertical extrusion of molars (mm)	Yes (Cochrane)
Xinyi Huang (a) (Huang et al. 2022)	2022	5	172	18	f/m	High expansion success; significant intermolar/alveolar width increases; greater skeletal expansion vs RPE; parallel suture opening; small relapse at 1 year; fewer periodontal side‐effects than RPE.	Intermolar width change (mm) before and after MARPE	Yes (Cochrane)
Xinyi Huang (b) (Huang et al. 2022)	2022	3	128	18.6	f/m		Alveolar ridge width (mm) pre‐ and post‐MARPE	Yes (Cochrane)
Xinyi Huang (c) (Huang et al. 2022)	2022	2	90	17.5	f/m		Intermolar width (mm) measured immediately and 1 year after MARPE	Yes (Cochrane)
Xinyi Huang (d) (Huang et al. 2022)	2022	2	90	17.5	f/m		Alveolar width (mm) immediately and 1 year after MARPE	Yes (Cochrane)
Xinyi Huang (e) (Huang et al. 2022)	2022	5	166	17.4	f/m		Tooth inclination of maxillary first molars post‐MARPE (degrees)	Yes (Cochrane)
Xinyi Huang (f) (Huang et al. 2022)	2022	3	148	16.7	f/m		Buccal alveolar bone height (mm) of maxillary first molars pre‐ and post‐MARPE	Yes (Cochrane)
Siddhisaributr (a) (Siddhisaributr et al. 2022)	2022	4	106	22	f/m	Pooled mean expansions—skeletal (e.g., PNS 3.34 mm, ANS 4.56 mm), alveolar (4.80 mm), dental (inter‐molar 5.99 mm); pyramidal pattern; successful expansion in late adolescents/adults.	Transverse width at zygomatic arches (mm)	Yes (Cochrane)
Siddhisaributr (b) (Siddhisaributr et al. 2022)	2022	9	191	23.1	f/m		Transverse width at nasal cavity (mm)	Yes (Cochrane)
Siddhisaributr (c) (Siddhisaributr et al. 2022)	2022	5	103	23.5	f/m		Transverse width at jugular notch (mm)	Yes (Cochrane)
Siddhisaributr (d) (Siddhisaributr et al. 2022)	2022	5	106	21.8	f/m		Posterior nasal spine expansion (mm)	Yes (Cochrane)
Siddhisaributr (e) (Siddhisaributr et al. 2022)	2022	5	106	21.8	f/m		Anterior nasal spine expansion (mm)	Yes (Cochrane)
Siddhisaributr (f) (Siddhisaributr et al. 2022)	2022	4	86	23.2	f/m		Alveolar ridge width at first molars (mm)	Yes (Cochrane)
Siddhisaributr (g) (Siddhisaributr et al. 2022)	2022	3	55	21.1	f/m		Intercanine transverse width (mm)	Yes (Cochrane)
Siddhisaributr (h) (Siddhisaributr et al. 2022)	2022	5	101	22.2	f/m		Interpremolar transverse width (mm)	Yes (Cochrane)
Siddhisaributr (i) (Siddhisaributr et al. 2022)	2022	9	167	22.3	f/m		Intermolar transverse width (mm)	Yes (Cochrane)
Jing Peng1 (a) (Peng et al. 2023)	2023	19	668	13–46	M/F	Mini screws decreased mandibular plane angle and intruded upper molars; no significant differences for lower molars, occlusal plane, SNB, chin position, or profile.	Mandibular plane angle change (degrees)	Yes (Cochrane)
Jing Peng1(b) (Peng et al. 2023)	2023	15	489	13–46	M/F		Vertical positional change of upper molars (mm)	Yes (Cochrane)
Jing Peng1 (c) (Peng et al. 2023)	2023	4	103	13–46	M/F		Vertical positional change of lower molars (mm)	Yes (Cochrane)
Jing Peng1 (d) (Peng et al. 2023)	2023	6	175	13–46	M/F		Occlusal plane angle change (degrees)	Yes (Cochrane)
Jing Peng1 (e) (Peng et al. 2023)	2023	13	422	13–46	M/F		Sagittal skeletal change (SNB angle)	Yes (Cochrane)
Jing Peng1 (f) (Peng et al. 2023)	2023	4	107	18–30	M/F		Anterior‐posterior position change of chin (mm)	Yes (Cochrane)
Jing Peng1 (g) (Peng et al. 2023)	2023	5	140	13–37	M/F		Soft tissue profile change (mm or degrees)	Yes (Cochrane)
LIU (a) (Liu et al. 2020)	2020	12	494	19.5	m/f	Mini screws reduced molar mesial movement (SMD − 1.48) and increased incisor retraction (SMD − 0.47) vs conventional anchorage; no significant vertical changes.	Mesiodistal displacement of molars (mm)	Yes (Cochrane)
LIU (b) (Liu et al. 2020)	2020	11	416	20	m/f		Mesiodistal displacement of incisors (mm)	Yes (Cochrane)
LIU (c) (Liu et al. 2020)	2020	6	232	21	m/f		Vertical displacement of molars (mm)	Yes (Cochrane)
LIU (d) (Liu et al. 2020)	2020	7	227	NR	m/f		Vertical displacement of incisors (mm)	Yes (Cochrane)
Yanhua Xu (a) (Xu and Xie 2017)	2017	11	376	NR	f/m	More incisor retraction (~1.5 mm) and less molar mesialization (~2.0 mm) with mini‐implants, a small SN–MP decrease (~1.1°), no differences in U1–SN/SNA, greater UL–E reduction and NLA increase, and no meaningful change in facial convexity.	SNA angle (sagittal position of maxilla)	Yes (Cochrane)
Yanhua Xu (b) (Xu and Xie 2017)	2017	13	413	NR	f/m		Maxillary incisor retraction (mm)	Yes (Cochrane)
Yanhua Xu (c) (Xu and Xie 2017)	2017	13	416	NR	f/m		Maxillary molar mesial or distal movement (mm)	Yes (Cochrane)

**Table 2:** Results of assess the methodological quality of meta‐analysis.

First author	Q1	Q2	Q3	Q4	Q5	Q6	Q7	Q8	Q9	Q10	Q11	Q12	Q13	Q14	Q15	Q16	Overall
Alharbi et al. (2019)	Yes	Yes	Yes	Partial Yes	Yes	Yes	Partial Yes	Partial Yes	Partial Yes	No	Yes	Yes	Yes	Yes	Yes	Yes	**Moderate**
Antoszewska‐Smith et al. (2017)	Yes	Yes	Yes	Partial Yes	Yes	No	Partial Yes	Partial Yes	Partial Yes	No	Yes	Yes	Yes	Yes	Yes	Yes	**Moderate**
Bi and Li (2022)	Yes	Yes	Yes	Partial Yes	No	No	Partial Yes	Partial Yes	Partial Yes	No	Yes	Yes	NO	Yes	NO	Yes	**Critially Low**
Peng et al. (2023)	Yes	Yes	Yes	Partial Yes	Yes	Yes	Partial Yes	Partial Yes	Yes	No	Yes	Yes	NO	Yes	Yes	Yes	**Low**
Kapetanović et al. (2021)	Yes	Yes	Yes	Partial Yes	Yes	Yes	Yes	Yes	Partial Yes	No	Yes	Yes	Yes	Yes	Yes	Yes	**High**
Liu et al. (2020)	Yes	Yes	Yes	Partial Yes	Yes	Yes	Yes	Yes	Yes	No	Yes	Yes	No	Yes	Yes	Yes	**Low**
Dos Santos et al. (2020)	Yes	Partial Yes	Yes	Partial Yes	Yes	Yes	Yes	Yes	Yes	No	Yes	Yes	Yes	Yes	Yes	Yes	**High**
Siddhisaributr et al. (2022)	Yes	Yes	Yes	Partial Yes	Yes	Yes	Yes	Partial Yes	Yes	No	Yes	Yes	no	no	Yes	no	**High**
Sosly et al. (2020)	Yes	Partial Yes	Yes	Partial Yes	Yes	Yes	Yes	Partial Yes	Yes	No	Yes	Yes	Yes	Yes	Yes	Yes	**Low**
Huang et al. (2022)	Yes	Yes	Yes	Partial Yes	no	Yes	Partial Yes	Yes	Yes	No	Yes	Yes	no	Yes	no	Yes	**Critially Low**
Xu and Xie (2017)	Yes	Partial Yes	Yes	Partial Yes	Yes	Yes	Yes	Partial Yes	Partial Yes	No	Yes	Yes	no	no	no	no	**Critially Low**

### 3.3 The Impact of Mini‐Screws on Inter‐Premolar Width

**Figure 2:**
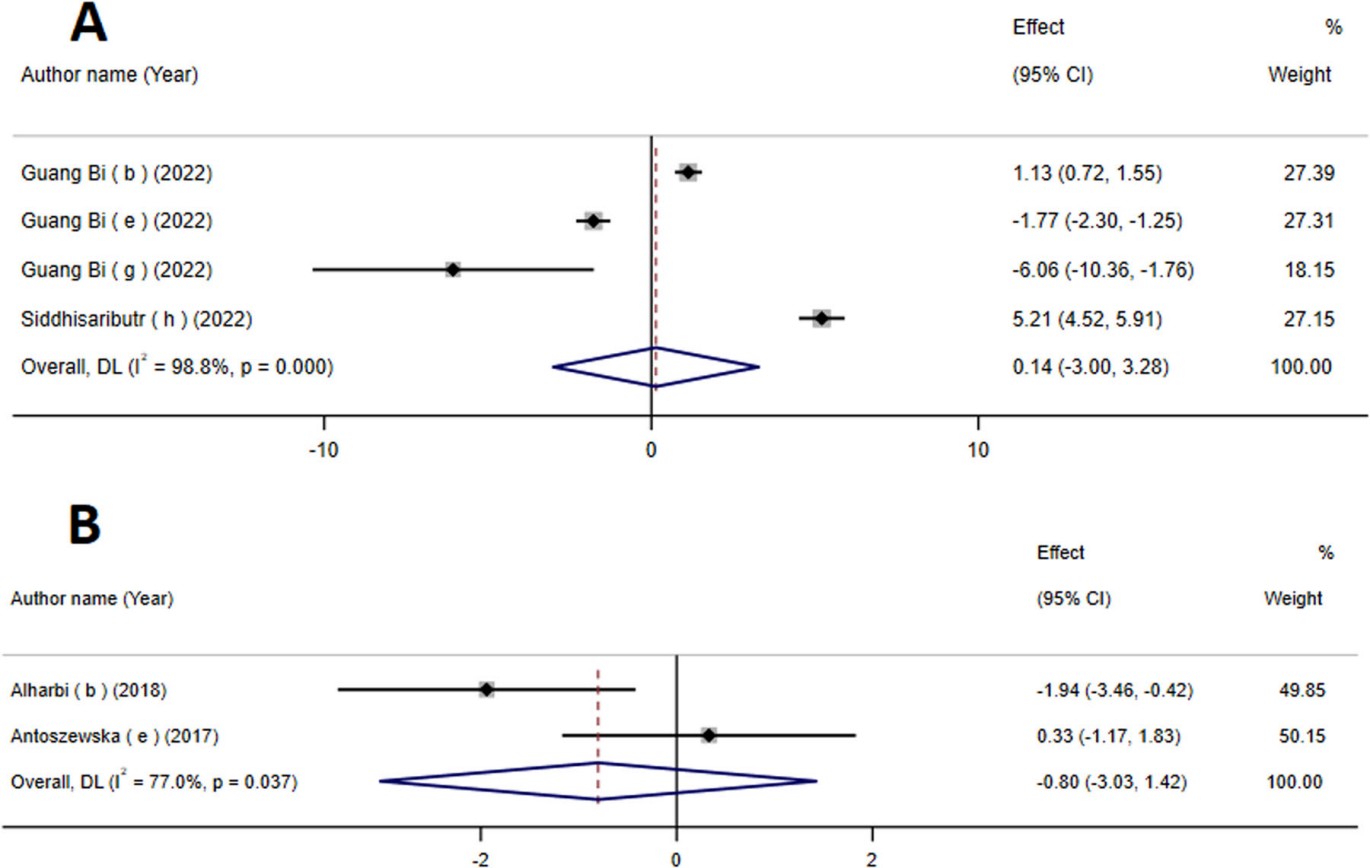
The effects of mini‐screws on inter‐premolar width (A) and treatment duration (B) detailing effect size (ES) and 95% confidence intervals (CIs).

Based on the analysis conducted by ESs, the integration of data from four meta‐analyses revealed lack of significant impact of mini‐screws on inter‐premolar width. (WMD:0.14; 95% CI: −3 to 3.28; *p* = 0.93) (Figure 2A). Moreover, a notable level of heterogeneity between studies was observed (*I* ^2^ = 98.8%, P‐heterogeneity < 0.001). No statistically significant difference was observed upon the exclusion of a single study using sensitivity analysis (Figure S1).

### 3.4 The Effect of Mini‐Screws on the Duration of Treatment

The findings indicate that the utilization of mini‐screws is associated with a decrease in treatment duration. However, it is important to note that these results lack statistical significance (SMD: −0.80; 95% CI: −3.03, 1.42; *p* = 0.48) (Figure 2B). Furthermore, a notable level of heterogeneity between studies was observed (*I* ^2^ = 77%, P‐heterogeneity = 0.03).

### 3.5 The Influence of Mini‐Screws on Inter‐Molar Width

**Figure 3:**
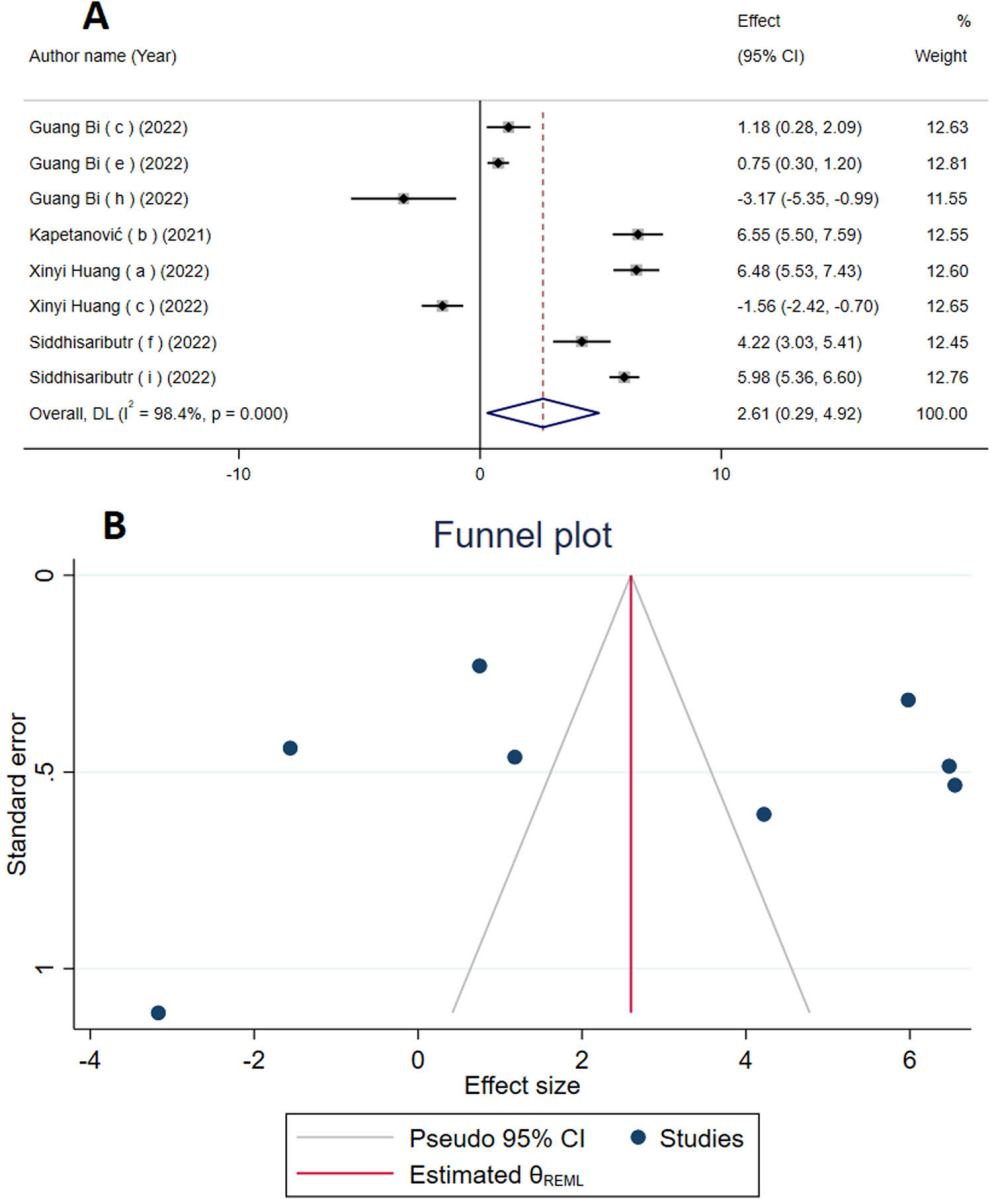
The Forest plot (A) and Funnel plot (B) of the effects of mini‐screws on inter‐molar width detailing effect size (ES) and 95% confidence intervals (CIs).

According to the analyses, it has been found that the utilization of mini‐screws can lead to a substantial increase in inter‐molar width (WMD = 2.61 mm; 95% CI: 0.29–4.92; *p* = 0.02) (Figure 3A). Moreover, a substantial level of heterogeneity between studies was observed (*I* ^2^ = 98.4%, P‐heterogeneity = 0.001). The subgroup analysis revealed that studies with extracted data sets of ≥ 5 demonstrated a more pronounced effect size on inter‐molar width compared to studies with data sets of < 5. These findings were statistically significant in both scenarios. Furthermore, upon conducting a subgroup analysis, it was observed that the studies employing palatal expansion as a treatment modality yielded statistically significant results. Conversely, the group utilizing maxillary expansion did not demonstrate statistically significant outcomes (Table 3). The statistical significance of the influence of mini‐screws on inter‐molar width was found to be altered following a sensitivity analysis that excluded the studies conducted by Kapetanović (b) (2021), Xinyi Huang (a) (2022), Siddhisaributr (f) (2022), and Siddhisaributr (i) (2022) (Figure S2). Funnel plots showed no evidence of asymmetry (Figure 3B), which was confirmed by Egger's and Begg's tests (*p* = 0.25 and *p* = 0.90, respectively).

**Table 3:** Subgroup analyses for the effects of mini‐screws and micro‐implants on orthodontic treatment outcomes.

	Effect size number	ES (95% CI)^a^	*p*‐within^b^	*I* ^2^ (%)^c^	P‐heterogeneity^d^
**Effect of Mini‐screw on inter‐molar width (MD)**					
Overall	8	2.61 (0.29, 4.92)	0.02	98.4%	< 0.001
**Number of included studies**					
< 5	4	1.10 (−0.69, 2.90)	0.421	95.1	< 0.001
≥ 5	4	4.23 (1.79, 6.67)	0.014	95.7	< 0.001
**Intervention**					
maxillary expansion	3	0.05 (−1.40, 1.50)	0.364	84.9	0.001
palatal expansion	5	4.33 (1.21, 7.46)	0.004	98.4	< 0.001
**Effect of Micro‐implants on movement of molars (MD)**					
Overall	4	−1.33 (−1.99, −0.26)	0.01	86.2	< 0.001
**Number of included studies**					
< 10	2	−0.31 (−0.88, 0.27)	0.134	0.0	0.5
≥ 10	2	−1.76 (−2.31, −1.21)	< 0.001	57.8	0.12

^a^ ES (95% CI): Effect size with corresponding 95% confidence interval.

^b^ P‐within: *P*‐value for the within‐group (subgroup) effect, indicating statistical significance of the pooled effect size.

^c^ *I* ^2^ (%): Measure of heterogeneity across studies, expressed as a percentage (0–100%). Higher values indicate greater heterogeneity.

^d^ P‐heterogeneity: *P*‐value from the Cochran's Q test assessing statistical significance of heterogeneity across included studies.

### 3.6 The Impact of Mini‐Screws on the Skeletal Width

**Figure 4:**
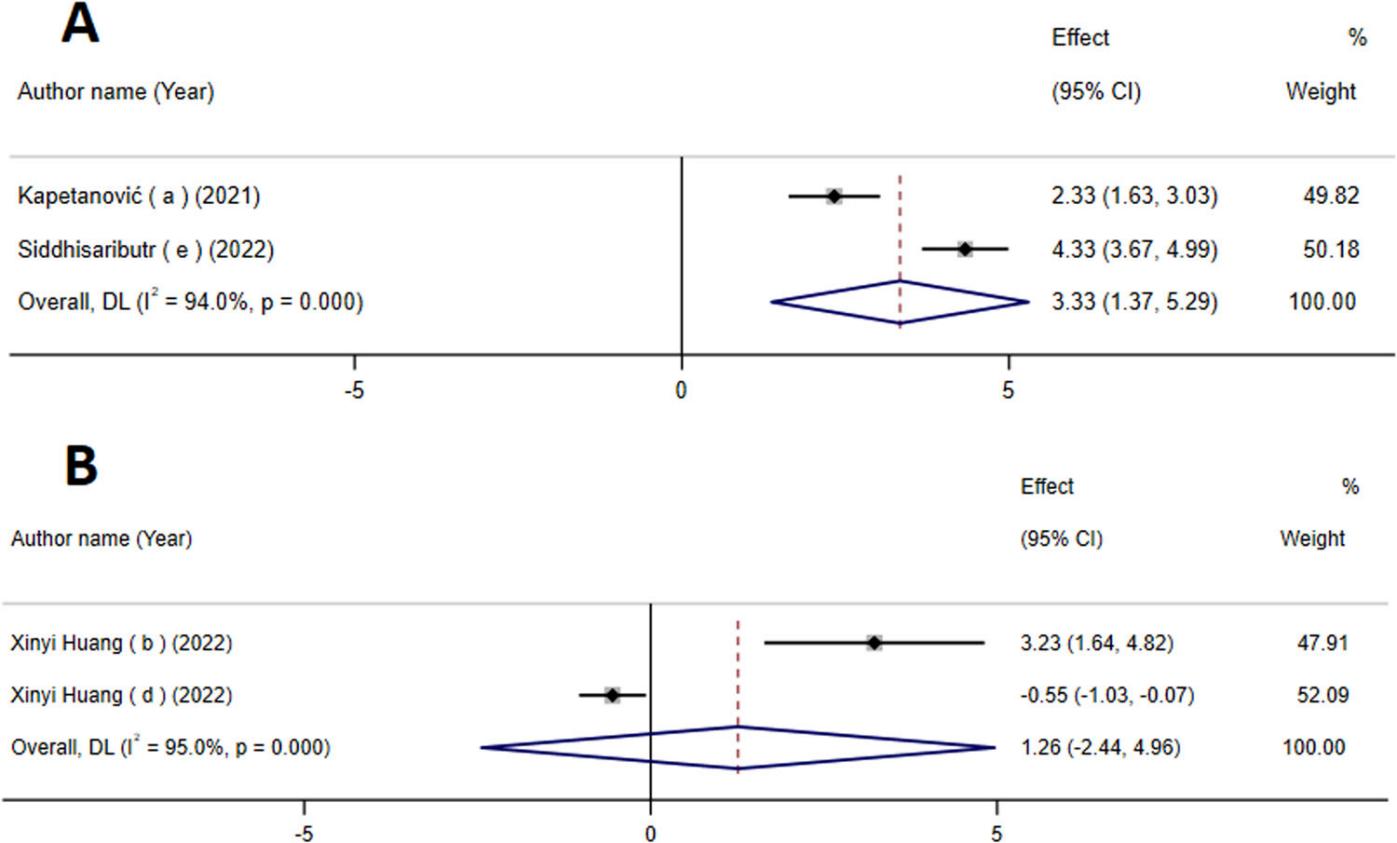
The effects of mini‐screws on skeletal width increase (A) and alveolar width before and after MARPE (B) detailing effect size (ES) and 95% confidence intervals (CIs).

The findings derived from the analysis of the data indicated that mini‐screws have a substantial impact on increasing skeletal width (WMD: 3.33 mm; 95% CI: 1.37–5.29; *p* = 0.001) (Figure 4A). However, it is important to note that there is a significant level of variation between studies (*I* ^2^ = 94%, *p* ≤ 0.001), indicating heterogeneity.

### 3.7 The Impact of Mini‐Screws on the Alveolar Width Before and After MARPE

The findings indicated that the use of mini‐screws did not have a significant impact on alveolar width both before and after the application of MARPE. (WMD: 1.26 mm, 95% CI: −2.44 to 4.96; *p* = 0.50) (Figure 4B). However, it is important to note that there was a significant level of heterogeneity among the studies. (*I* ^2^ = 95%, P‐heterogeneity ≤ 0.001).

### 3.8 The Effects of Mini‐Screws on Tooth Movement

**Figure 5:**
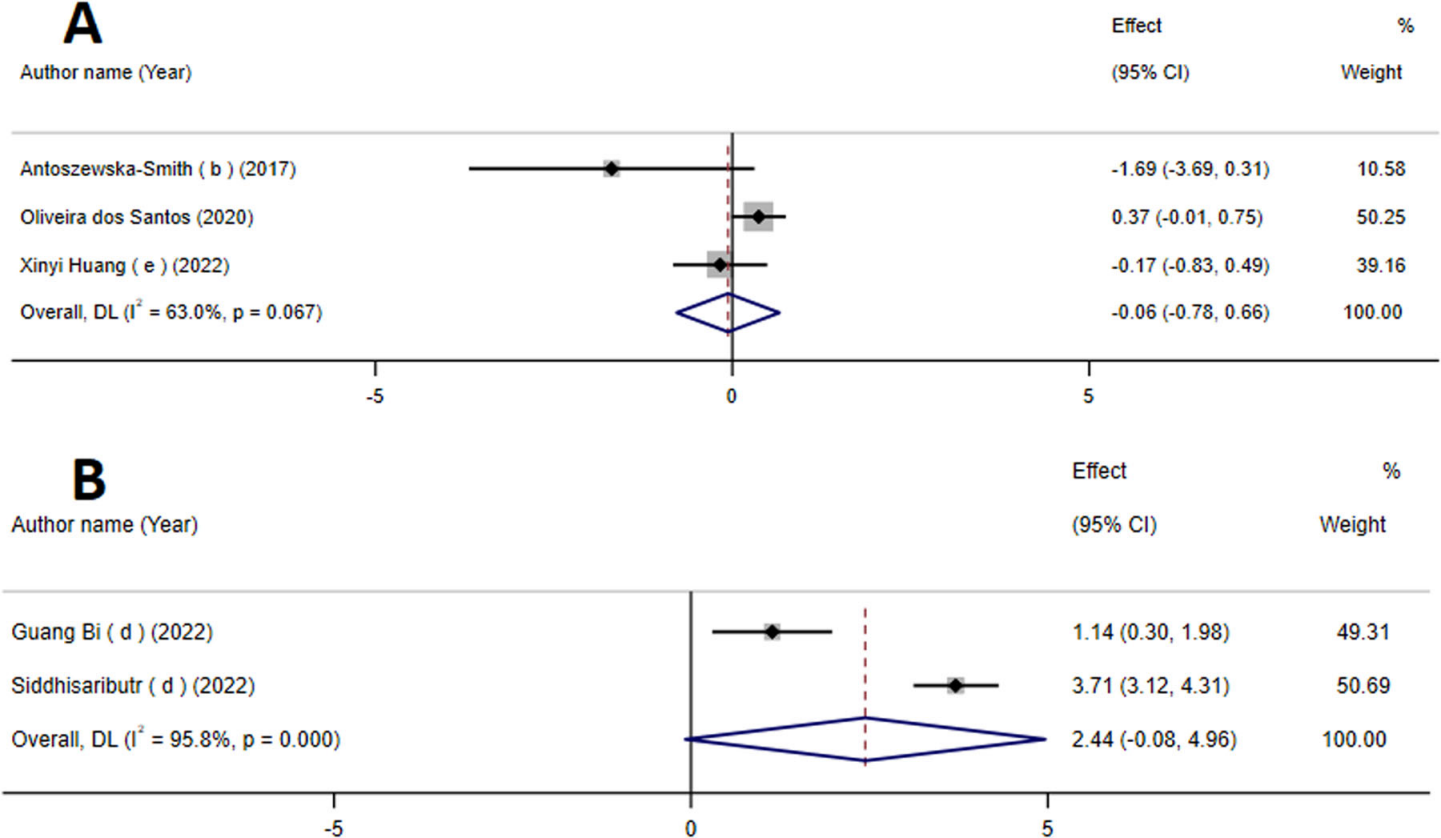
The effects of mini‐screws on tooth movement (A) and mid‐palatal suture expansion (B) detailing effect size (ES) and 95% confidence intervals (CIs).

The findings suggest that the use of mini‐screws does not have a substantial impact on tooth movement (WMD: −0.06 mm; 95% CI: −0.78, 0.66, *p* = 0.87; based on three meta‐analyses) (Figure 5A). Additionally, there was notable variability between studies (*I* ^2^ = 63%, P‐heterogeneity = 0.06).

### 3.9 The Effect of Mini‐Screws on the Expansion of the Mid‐Palatal Suture

The findings indicate that the use of mini‐screws did not have a statistically significant impact on the expansion of the mid‐palatal suture (WMD: 2.44 mm; 95% CI: −0.08, 4.96; *p* = 0.09) (Figure 5B). Additionally, there was a notable level of heterogeneity observed between the studies (*I* ^2^ = 95.8%, *p* < 0.06).

### 3.10 The Effect of Micro‐Implants on the Movement of Molars

**Figure 6:**
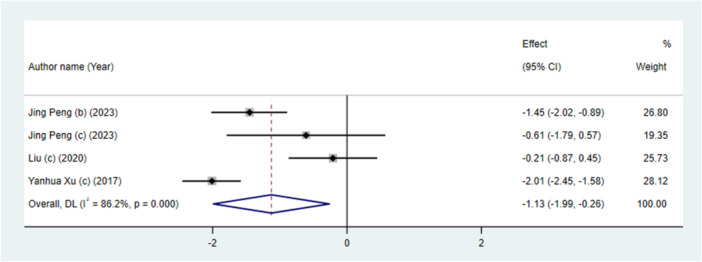
The effects of micro‐implants on movement of molars detailing effect size (ES)and 95% confidence intervals (CIs).

Combining the data from four meta‐analyses indicated a significant effect of micro‐implants on the movement of molars (WMD: −1.13 mm; 95% CI: −1.99, −0.26, *p* = 0.01) (Figure 6) with significant between‐study heterogeneity (*I* ^2^ = 86.2%, P‐heterogeneity < 0.001) which was reduced when subgrouping by the number of included studies (Table 3). The statistical significance of the influence of mini‐screws on inter‐molar width was altered following a sensitivity analysis with the exclusion of Jing Peng (b) (2023) and Yanhua Xu (c) (2017) (Figure S4).

### 3.11 Qualitative Summary of Non‐pooled Studies

The study conducted by Antoszewska‐Smith in 2017 in Poland (Antoszewska‐Smith et al. 2017) investigated the mesial movement of molars. The findings of the study demonstrated that the utilization of mini‐screws resulted in a significant reduction in molar movement. Additionally, a study conducted by Alharbi (a) in 2018 (Alharbi et al. 2019) within the context of Saudi Arabia demonstrated that the utilization of mini‐screws has a noteworthy impact on reducing anchorage loss. The Antoszewska‐Smith study (Antoszewska‐Smith et al. 2017) examined the effects of mini‐screws on the retraction and tipping of incisors. The findings indicate that mini‐screws lead to a notable reduction in incisor retraction, but do not have a significant impact on incisor tipping. In a study conducted by Sosly in 2020 within the United Kingdom (Sosly et al. 2020), the impact of mini‐screws on three specific outcomes was examined. These outcomes included the extent of deep‐bite correction, the degree of incisor intrusion (Cr‐PP), and the level of molar extrusion. The findings of the study demonstrated statistical significance across all three aforementioned cases. Based on the findings of a study conducted by Xinyi Huang in 2022 in China (Huang et al. 2022), it has been observed that mini‐screws have exerted a notable influence on the buccal alveolar height of both the right and left maxillary first molars. The study conducted by Siddhisaributr et al. (2022) examined the impact of mini‐screws on the increase of zygomatic width, nasal width, and jugular width. The findings revealed a significant effect of mini‐screws on the augmentation of these facial measurements. In the research conducted by Peng et al. (2023), an examination was conducted to assess the impact of micro‐implants on five specific outcomes. These outcomes included alterations in the mandibular plane, occlusal plane, the angle between the Sella/nasion plane and nasion/B plane (SNB) angle, chin position, and profile changes. The findings were statistically significant solely for the variable of change of mandibular plane and, vertical change of upper molar, while no statistically significant results were observed for the other outcomes. Based on the findings of Liu's study, it has been observed that the utilization of micro‐implants leads to a notable enhancement in the mesiodistal movements of molars and incisors. However, it has been determined that these micro‐implants do not exert a substantial impact on the vertical movement of incisors. Furthermore, the findings of Yanhua Xu's research (Xu and Xie 2017) indicate that the utilization of micro‐implants leads to a notable reduction in maxillary incisor retraction. However, it does not yield a significant impact on the angle between the Sella/nasion plane and the nasion/A plane (SNA) variable.

## 4 Discussion

Our analysis shows that mini‐screws significantly increase intermolar and skeletal width, though their impact on other outcomes is not statistically significant. Additionally, micro‐implants were found to significantly reduce molar movement. This umbrella meta‐analysis aggregates findings from existing systematic reviews and meta‐analyses to provide a comprehensive evaluation of orthodontic anchorage outcomes.

The analysis revealed that mini‐screws did not have a statistically significant effect on interpremolar width. However, it is noteworthy that individual studies included in the analysis reported statistically significant results for this outcome (Siddhisaributr et al. 2022; Bi and Li 2022). One possible contributing factor is the variability in the number of included studies and the differing sample sizes of the intervention groups, leading to a wide range of outcomes. Another factor may be the differences in the types of interventions applied. The Bi and Li (2022) data sets employ intervention minis crew‐assisted rapid maxillary expansion (MARME), whereas the Siddhisaributr (h) (Siddhisaributr et al. 2022) study utilizes a minis crew‐assisted rapid palatal expansion (MARPE). In MARME, small screws are placed in the maxilla to achieve palate expansion, while in MARPE, mini‐screws are inserted into the palate to facilitate maxillary widening. Another factor influencing the observed outcomes is the variation in study quality—three studies were rated as critically low, while one was rated high, potentially contributing to inconsistencies in the findings. Additionally, mini‐screws did not significantly affect treatment duration, possibly due to the nature of the intervention. Unlike Temporary Anchorage Devices (TADs), which are temporarily affixed to the bone and easily removable, mini‐screws are mechanically secured in the bone and remain in place throughout treatment, relying on their design and structure for retention (Mizrahi and Mizrahi 2007). Furthermore, the health status of the patients can be identified as a contributing factor. This is evident in a study conducted by Alharbi (b), (Alharbi et al. 2019), where the increased severity of the disease resulted in a notable impact of mini‐screws on variable treatment duration. The analysis concludes that the use of mini‐screws significantly increases intermolar width. Mini‐screws are typically placed in the buccal shelf area or the inter‐radicular space between the molars, serving as anchor points to apply force that gradually moves the molars apart. This force triggers remodeling of the periodontal ligament, causing bone resorption on the compression side and bone deposition on the tension side, thereby facilitating the gradual expansion of the intermolar space (Kim et al. 2010). Also, the results of all the included meta‐analyses showed a significant increase in inter‐molar width except for Xinyi Huang (c) study (Huang et al. 2022). A possible explanation for the decreased intermolar width in this study is that the measurement was taken 1‐year posttreatment, unlike in other studies. Additionally, subgroup analysis revealed that studies employing palatal expansion methods reported a greater increase in intermolar width.

The findings also showed that mini‐screws significantly increase skeletal width, likely due to their role in orthopedic expansion. This process involves applying forces to the bones to stimulate growth and widen the skeletal structure. Mini‐screws provide stable anchorage for appliances such as palatal expanders or facemasks, enabling precise force application to the maxilla or mandible without affecting adjacent teeth (Solano Mendoza et al. 2022).

The results indicated that mini‐screws did not significantly affect alveolar width before or after MARPE, despite individual studies showing significant results. This discrepancy may be due to the limited number of studies and small sample sizes in the overall analysis. Similarly, the small sample size may explain the lack of a significant effect of mini‐screws on mid‐palatal suture expansion. Mini‐screws also showed no substantial impact on tooth movement. Possible reasons for this include improper placement, insufficient stability, inadequate force application, or poor patient compliance with orthodontic instructions. Additionally, factors such as bracket placement, archwire selection, and overall treatment planning play a critical role in influencing tooth movement, as mini‐screws are only one component of the orthodontic approach (Topouzelis and Tsaousoglou 2012).

The results indicate that micro‐implants significantly reduce molar movement. By being anchored into the bone, micro‐implants provide a stable point for applying orthodontic forces. In some cases, they help prevent or minimize unwanted molar movement caused by external forces, such as pressure from the tongue or cheeks (Chen et al. 2006). Micro‐implants provide a stable anchorage point, allowing the orthodontist to counteract unwanted forces and prevent further molar movement. Traditional orthodontic methods often use adjacent teeth as anchor points, which can lead to undesirable movement or tipping of those teeth. By using micro‐implants, reliance on other teeth for anchorage is reduced, minimizing these unwanted side effects (Melsen and Costa 2000). On the other hand, the results of two studies Jing Peng (c) and Liu (c) (Peng et al. 2023; Liu et al. 2020) are not significant, while other studies have shown a significant effect. The possible reason for which is the low sample size of these two studies compared to other studies.

The results of several data sets analysis showed that the use of mini‐screws can reduce the outcomes of movements of molars, anchorage loss, and retraction of incisors during treatment (Alharbi et al. 2019; Antoszewska‐Smith et al. 2017). Mini‐screws provide direct bone anchorage, stabilizing molars during anterior tooth retraction. They also create a counteracting force system to maintain molar alignment, enable stronger orthodontic forces for efficient tooth movement, and help reduce treatment time by minimizing the need for additional devices (Papadopoulos and Papageorgiou 2013; Park et al. 2006).

The findings of data sets analysis indicate that the utilization of micro‐implants has demonstrated a reduction in outcomes such as mandibular plane and vertical changes of movement of molars during orthodontic treatment (Peng et al. 2023; Liu et al. 2020). These outcomes can be attributed to similar factors as observed in previous cases. Furthermore, the study conducted by Lee et al. (2022) corroborated the results obtained in the present study (Lee et al. 2022).

### 4.1 Limitation and Strength

This review has several notable strengths, including a comprehensive synthesis of findings from multiple meta‐analyses on the effects of mini‐screws and micro‐implants in orthodontic treatment. Notably, it is the first umbrella review conducted in this field, as confirmed by the search results. However, the study also has limitations, such as the small number of included studies and limited sample sizes for many outcomes. Significant heterogeneity, which could not be fully explained through subgroup analyses, further limits the findings. Additionally, while methodological quality was assessed using AMSTAR 2, the internal validity of the clinical trials within the meta‐analyses was not evaluated, potentially affecting the reliability of the pooled estimates. Another important methodological consideration is our treatment of heterogeneity. Following the Cochrane Handbook, we considered I² values greater than 75% to indicate high heterogeneity. In such cases, results were not excluded from the umbrella review but were interpreted qualitatively rather than quantitatively pooled. This approach minimizes the risk of producing misleading summary estimates; however, it also reduces the number of outcomes available for statistical synthesis. Consequently, some findings were underrepresented in the quantitative analyses, underscoring the need for cautious interpretation. Future systematic reviews with more homogeneous study designs and standardized outcome measures are required to provide more robust pooled estimates. Further research is needed to address these limitations and enhance evidence in this area.

## 5 Conclusion

In conclusion, this study demonstrated that mini‐screws and micro‐implants has a positive and significant effect on inter‐molar width, ANS, and movement of molars in the treatment process of patients during orthodontic treatment, and it seems that they can be used as a treatment option in different conditions.

## Author Contributions

**Abdolreza Jamilian:** drafting of the manuscript, acquisition of data. **Kurosh Majidi:** drafting of the manuscript, acquisition of data. **Helen Jamloo:** drafting of the manuscript, acquisition of data, critical revision of the manuscript for important intellectual content. **Meysam Zarezadeh:** interpretation of data, critical revision of the manuscript for important intellectual content, study concept and design, content, study concept and design.

## Conflicts of Interest

The authors declare no conflicts of interest.

## Supporting information

supporting file 1.

supporting file 2.

supporting file 3.

PRISMA 2020 checklist.

## Data Availability

Data will be made available at reasonable request.
