# Supplementary material for: The Impact of Mini‐Screws and Micro‐Implants on Orthodontic Clinical Outcomes: An Umbrella Meta‐Analysis
Source: Clin Exp Dent Res. 2025 Sep 8;11(5):e70220. doi: 10.1002/cre2.70220 (PMC12415713; doi:10.1002/cre2.70220)
Supplement: Supplementary file 1 — supporting file 1. [file CRE2-11-e70220-s002.docx]

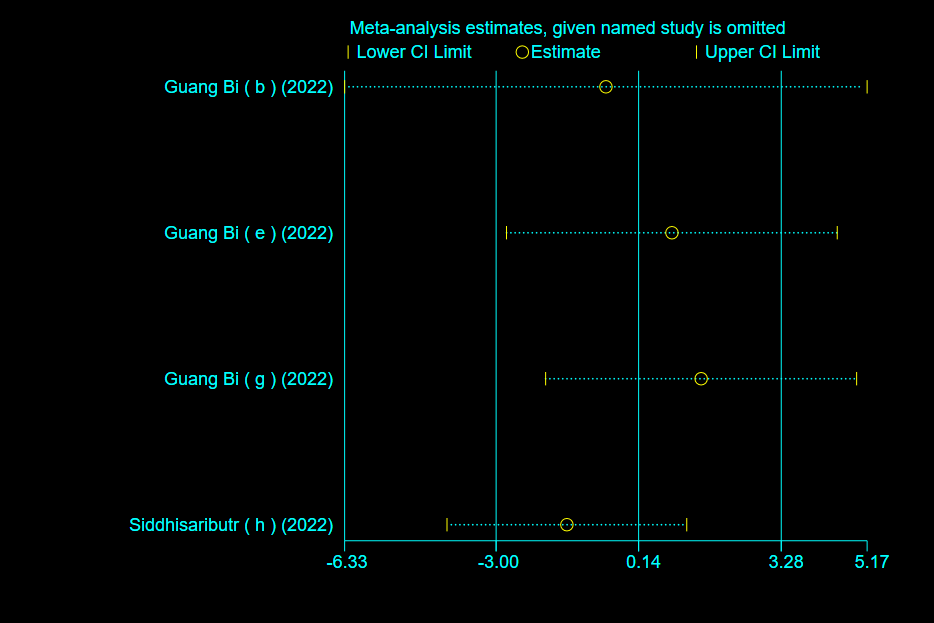


Sensitivity analysis of the impact of mini-screws on inter-premolar width


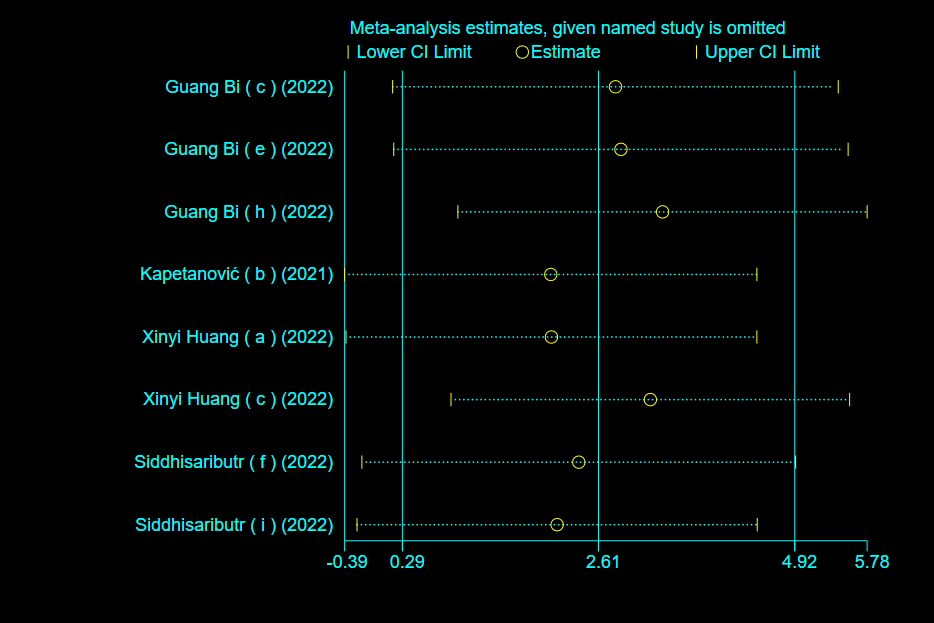


Sensitivity analysis of the influence of mini-screws on inter-molar width


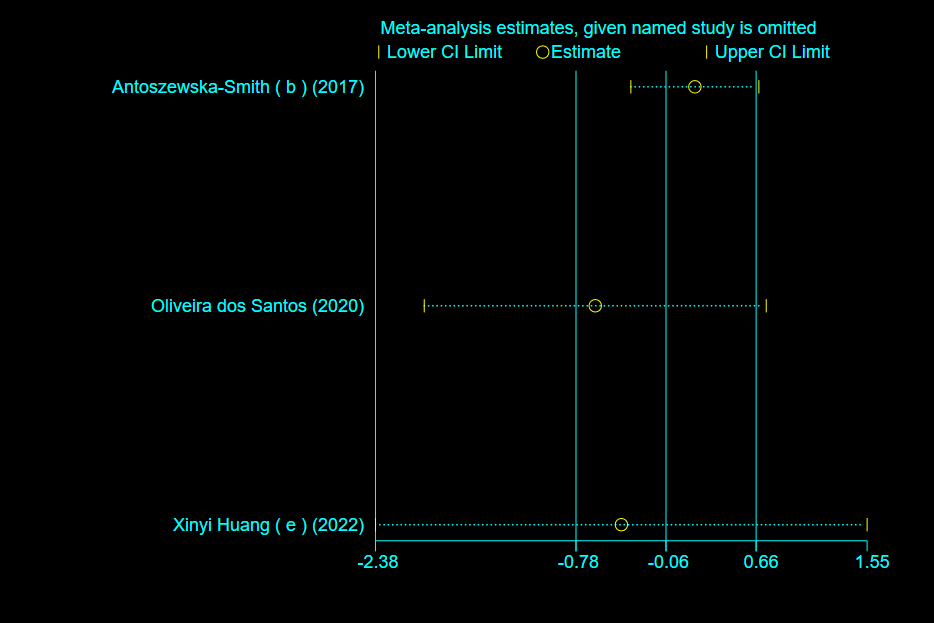


Sensitivity analysis of the effect of the use of mini-screws on the expansion of the mid-palatal suture


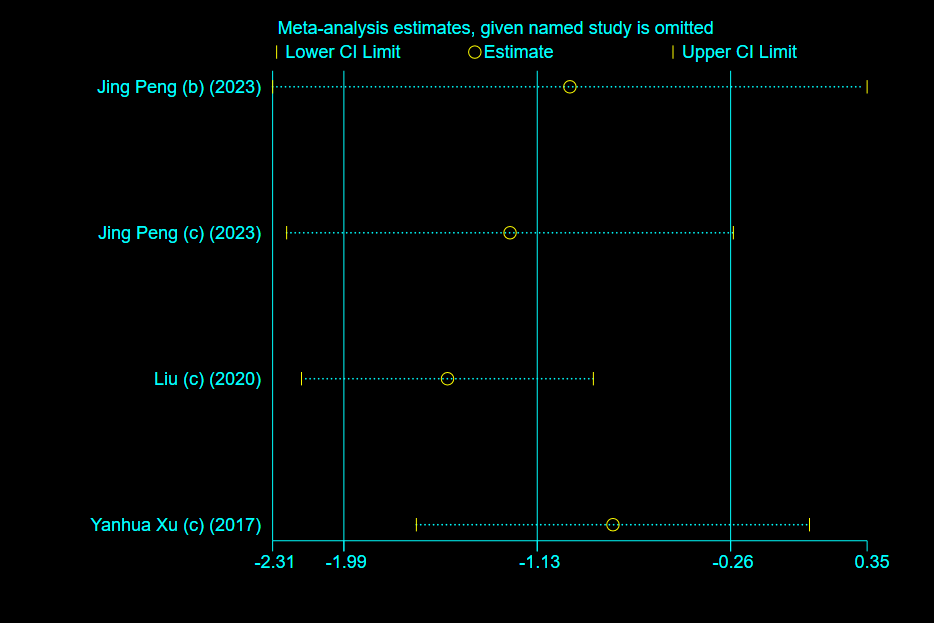


Sensitivity analysis of the effect of micro-implants on the movement of molars
