# Supplementary material for: The Impact of Mini‐Screws and Micro‐Implants on Orthodontic Clinical Outcomes: An Umbrella Meta‐Analysis
Source: Clin Exp Dent Res. 2025 Sep 8;11(5):e70220. doi: 10.1002/cre2.70220 (PMC12415713; doi:10.1002/cre2.70220)
Supplement: Supplementary file 2 — supporting file 2. [file CRE2-11-e70220-s001.docx]

**Search strategy of databases**

**MEDLINE (via PubMed):**

("Orthodontics"[MeSH Terms] OR "Malocclusion"[MeSH Terms] OR orthodontic*[Title/Abstract] OR malocclusion*[Title/Abstract] OR "braces"[Title/Abstract] OR "tooth movement"[Title/Abstract])

AND

("Dental Implants"[MeSH Terms] OR "mini-screw"[Title/Abstract] OR "mini screw"[Title/Abstract] OR "mini-implant"[Title/Abstract] OR "micro-implant"[Title/Abstract] OR "temporary anchorage device"[Title/Abstract] OR TAD[Title/Abstract])

AND

("Meta-Analysis as Topic"[MeSH Terms] OR "meta-analysis"[Publication Type] OR "systematic review"[Title/Abstract] OR "meta-analysis"[Title/Abstract] OR meta[Title/Abstract])

**Scopus:**

(TITLE-ABS-KEY(orthodontic* OR malocclusion* OR "braces" OR "tooth movement"))

AND

(TITLE-ABS-KEY("mini-screw" OR "mini screw" OR "micro screw" OR "micro-implant" OR "mini implant" OR "temporary anchorage device" OR TAD))

AND

(TITLE-ABS-KEY("meta-analysis" OR "systematic review" OR meta))

**Web of Science:**

TS=(orthodontic* OR malocclusion* OR "braces" OR "tooth movement")

AND

TS=("mini-screw" OR "mini screw" OR "micro screw" OR "micro-implant" OR "mini implant" OR "temporary anchorage device" OR TAD)

AND

TS=("meta-analysis" OR "systematic review" OR meta)

**Google Scholar:**

(Used manually as a supplementary check using keywords: "Orthodontics", "mini-screws", "micro-implants", "meta-analysis")
