# Supplementary material for: The Impact of Mini‐Screws and Micro‐Implants on Orthodontic Clinical Outcomes: An Umbrella Meta‐Analysis
Source: Clin Exp Dent Res. 2025 Sep 8;11(5):e70220. doi: 10.1002/cre2.70220 (PMC12415713; doi:10.1002/cre2.70220)
Supplement: Supplementary file 3 — supporting file 3. [file CRE2-11-e70220-s004.docx]

| **Supplementary Table.** List of Full-Text Articles Excluded After Screening with Reasons for Exclusion (PRISMA 2020 Item 16b | | | |
| --- | --- | --- | --- |
| **Author(s)** | **Year** | **Title (Short)** | **Reason for Exclusion** |
| Papageorgiou et al. | 2012 | Failure rates of orthodontic miniscrew implants | Focused on failure rate only; not reporting therapeutic outcomes |
| Marques de Mattos et al. | 2022 | Stability of mini-implants and mini-plates | Compared mini-implants vs. mini-plates; not aligned with PICOs |
| Papadopoulos et al. | 2011 | Clinical effectiveness of miniscrew implants | Focused on implant survival and anchorage success rate only |
| Tepedino et al. | 2020 | Interradicular site & cortical thickness | Observational study; did not assess treatment outcomes |
| Lee et al. | 2020 | Cortical thickness and success rates | Focused on risk factors (bone density); not treatment outcomes |
| Casana-Ruiz et al. | 2020 | Risk factors for biological stability | Risk factor analysis only; not a treatment efficacy review |
| Sheibaninia et al. | 2020 | Failure rate and associated factors | Focused on failure risks; not pooled treatment outcomes |
| Marquezan et al. | 2014 | Cortical thickness and primary stability | Observational prognosis study; outcome not therapeutic |
| Al-Thomali et al. | 2022 | Surface treatment and mechanical stability | Focus on implant surface mechanics, not treatment outcome |
| Papageorgiou et al. | 2022 | Periodontal–orthodontic treatment in periodontitis | Mixed interventions; does not focus on mini-screws/micro-implants |
| Jedliński et al. | 2021 | Guided insertion of TADs with 3D templates | Focus on insertion technique accuracy, not clinical outcomes |
| Jedliński et al. | 2022 | Thread design and stability of mini-implants | Focus on implant thread geometry/stability, not clinical outcomes |
| Li et al. | 2020 | MI vs. conventional anchorage in dentoalveolar protrusion | Mixed observational and RCTs; inconsistent with PICO intervention scope |
| Wang et al. | 2020 | Protocol for Class II correction with micro-implants | Protocol only; no outcome data or meta-analysis results |
| Mohammed et al. | 2018 | Anatomical site & risk factors for failure | Focus on failure rates by site; does not report therapeutic outcomes |
| Devadkar et al. | 2022 | MI success based on jaw and insertion side | Focus on success rate variation (site/jaw), not treatment outcomes |
| Yi et al. | 2016 | Self-drilling vs. self-tapping miniscrew success rates | Focus on insertion technique/stability only — no clinical effect outcomes |
